# Effectiveness of toric intraocular lens implantation for correcting irregular corneal astigmatism in cataract eyes

**DOI:** 10.1038/s41598-024-59303-0

**Published:** 2024-04-17

**Authors:** Xiteng Chen, Yuanfeng Jiang, Nan Gao, Yichen Gao, Jun Yang, Shaochong Bu, Fang Tian

**Affiliations:** Tianjin Key Laboratory of Retinal Functions and Diseases, Tianjin Branch of National Clinical Research Center for Ocular Disease, Eye Institute and School of Optometry, Tianjin Medical University Eye Hospital, 251 Fukang Road, Tianjin, 300000 China

**Keywords:** Lens diseases, Refractive errors

## Abstract

A retrospective cohort study was conducted to observe the correction effect of Toric intraocular lens (IOL) implantation in cataract eyes with specific types of irregular corneal astigmatism. Thirty-four eyes with either the "asymmetric bow-tie" pattern (Type I) or the "angled bow-tie" pattern (Type II) were included. Corneal topography was assessed using Pentacam HR, and changes in preoperative corneal astigmatism, visual acuity, manifest refraction, and objective visual quality were measured and compared. The average uncorrected distance visual acuity improved significantly from 0.86 ± 0.40 logMAR to 0.22 ± 0.15 logMAR (P < 0.001). Preoperative corneal astigmatism of 2.05 ± 0.90 D was corrected to a postoperative residual astigmatism of 0.78 ± 0.57 D (P < 0.001), with 32% of eyes within 0.50 D. The residual astigmatism prediction errors in Type I and Type II cases were (0.97 ± 0.68 D) and (0.66 ± 0.37 D), respectively (P = 0.100). The mean spherical equivalent prediction error in Type II cases (0.07 ± 0.36 D) was significantly smaller than that in Type I cases (− 0.29 ± 0.52 D) (P = 0.030). This study concludes that Toric IOL implantation effectively corrects specific types of irregular corneal astigmatism in cataract surgery. Eyes with the "angled bow-tie" pattern show higher accuracy in refractive predictions compared to eyes with the "asymmetric bow-tie" pattern.

## Introduction

Residual astigmatism after cataract surgery directly compromises the visual acuity and leads to glare, monocular diplopia and visual distortions, even at low degree^1^. Minimizing astigmatism is crucial for enhancing postoperative visual quality. Toric intraocular lenses (IOL) have emerged as a widely adopted option in cataract surgery, offering an effective and dependable solution for patients with regular corneal astigmatism^2,3^. However, for some eyes, irregular corneal astigmatism poses challenges in vision correction, leading to decreased visual acuity, reduced quality of life, and lower patient satisfaction^4^.

Irregular astigmatism is typically caused by keratoconus, pterygium, corneal degeneration, corneal scar, and post-keratoplasty^5^. Theoretically, conventional cylindrical lenses can only correct regular astigmatism. Certain eyes with irregular astigmatism could only achieve further improvement through limited and invasive refractive correction methods, such as corneal laser surgery and postoperative corneal contact lenses^5^. Today, treating the surface of irregular corneas with customized photoablation using an excimer laser is possible, aiming to eliminate high-order aberrations and expand the optical zone^6^. In recent years, some experts have broken the contraindication to use Toric IOL in such irregular cases, seeking to neutralize the low-order astigmatism component of preoperative corneal aberrations^7–9^. Despite this, its effectiveness and indicating scenarios remain uncertain.

Hence, this study sought to investigate the treatment potential and compare the effectiveness of Toric IOL implantation in two specific types of irregular astigmatism cases.

## Results

A total of 36 patients (36 eyes) were enrolled in this study. After excluding two patients who were unable to complete the one-month follow-up, the final statistical analysis included 34 eyes. The demographic characteristics and relevant preoperative data are shown in Table 1. No complications were observed in any of the cases. The average UCVA and BCVA improved from 0.86 ± 0.40 logMAR and 0.29 ± 0.21 logMAR to 0.22 ± 0.15 logMAR and 0.11 ± 0.14 logMAR, respectively (*t* = 8.665 and 4.067, both *P* < 0.001).

**Table 1:** Demographic characteristics of the patients and relevant preoperative data.

Parameter	Total	Type I	Type II
Patients/eyes (n)	34/34	14/14	20/20
Female/male (n)	22/12	9/5	13/7
Age (years)	72.65 ± 12.31	75.29 ± 8.53	70.80 ± 12.80
OD/OS (n)	19/15	6/8	13/7
WTR/ATR/Oblique (eyes)	6/22/6	2/7/5	4/15/1
IOL master 700 results			
Axial length (mm)	23.06 ± 0.97	22.75 ± 0.92	23.27 ± 0.97
Anterior chamber depth (mm)	2.79 ± 0.41	2.93 ± 0.50	2.70 ± 0.38
Cornea thickness (mm)	527.29 ± 39.33	520.50 ± 49.78	532.05 ± 30.55
White-to-white (mm)	11.50 ± 0.47	11.44 ± 0.56	11.55 ± 0.41
K1 (flat) (D)	43.82 ± 1.70	44.50 ± 1.68	43.35 ± 1.60
K2 (steep) (D)	45.87 ± 1.96	46.87 ± 2.27	45.17 ± 1.37
ΔK (D)	2.05 ± 0.90 (1.08 ~ 5.24)	2.37 ± 1.15 (1.15 ~ 5.24)	1.82 ± 0.59 (1.08 ~ 3.03)
Pentacam results			
HOAs	0.577 ± 0.449	0.733 ± 0.589	0.468 ± 0.288
Irregularity	0.066 ± 0.043	0.077 ± 0.049	0.058 ± 0.038
ISV	32.71 ± 14.83	38.07 ± 16.99	28.95 ± 12.19
IOL			
Spherical equivalent (D)	21.87 ± 2.75	21.71 ± 3.47	21.98 ± 2.20
Cylinder (T3/T4/T5/T6/T7/T8/T9)	6/8/13/3/2/0/2	2/2/7/1/0/0/2	4/6/6/2/2/0/0

*SD* standard deviation, *WTR* with the rule astigmatism, *ATR* against the rule astigmatism, *K* standard keratometry, *D* diopters, *HOAs* high order aberrations, *ISV* index of surface variance.

### Preoperative corneal astigmatism and residual astigmatism

**Figure 1:**
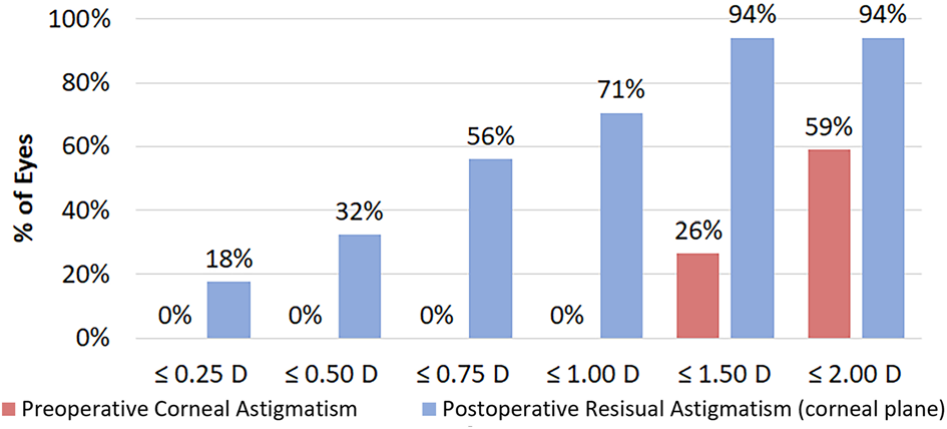
The distribution of preoperative corneal astigmatism and postoperative residual astigmatism. *D* diopters.

**Figure 2:**
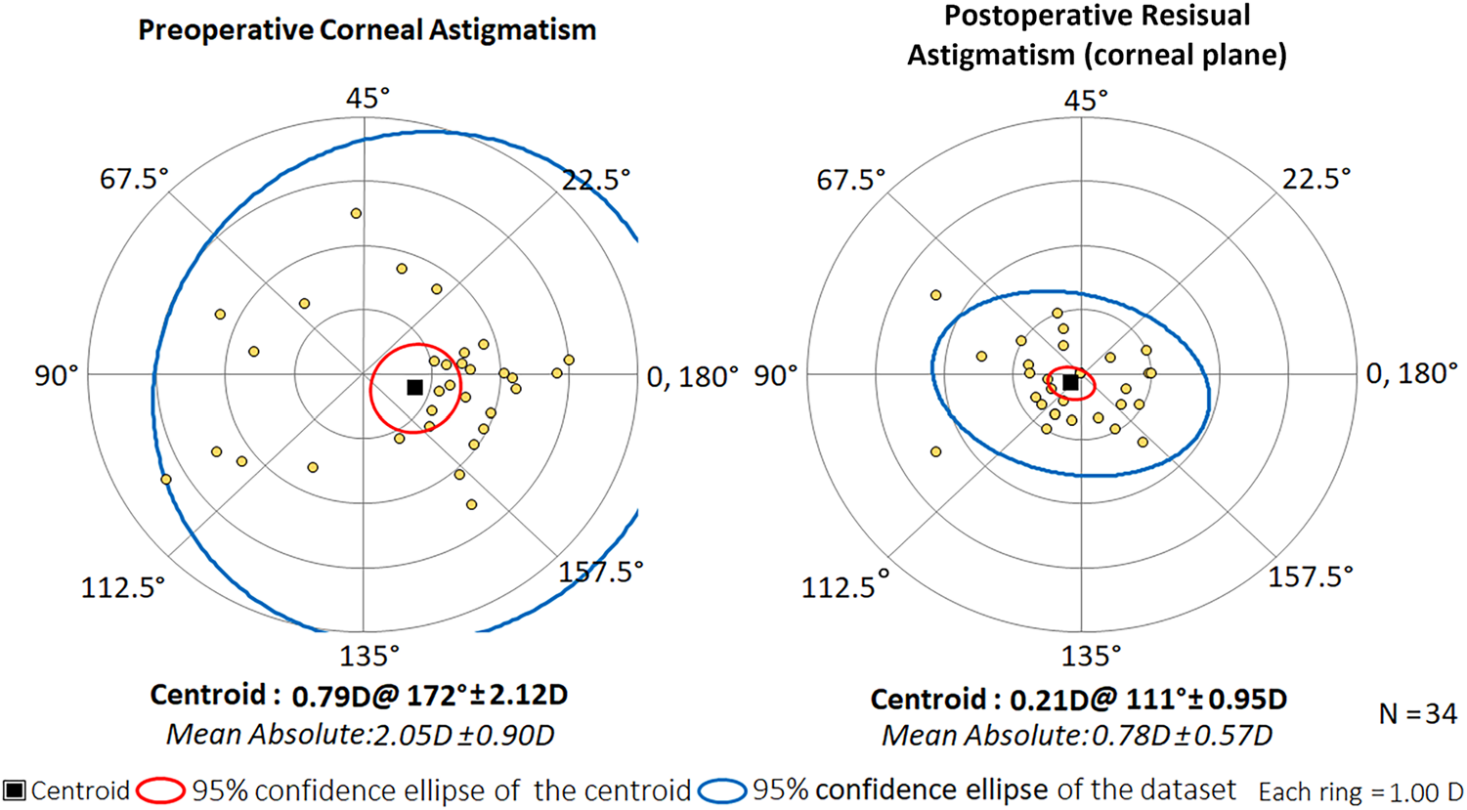
Double-angle plots of preoperative corneal astigmatism and postoperative residual astigmatism. The mean residual astigmatism (corneal plane, 0.78 ± 0.57 D) was significantly decreased compared to the preoperative mean corneal astigmatism (2.05 ± 0.90 D) (*t* = 10.320, *P* < 0.001). *D* diopters.

The residual astigmatism (converted from the postoperative manifest refraction to the corneal plane) at one month postoperatively was 0.78 ± 0.57 (range: 0 to 2.43) D, which significantly decreased compared to the preoperative mean corneal astigmatism of 2.05 ± 0.90 D (range: 1.08–5.24) D (*t* = 10.320, *P* < 0.001). The average reduction in astigmatism magnitude was 1.27 ± 0.72 D (range: 0.09–2.81 D). Postoperatively, 32% of the patients had a residual astigmatism below 0.50 D, while 71% had that of less than 1.00 D (Fig. 1). Figure 2 illustrates the distribution of astigmatic vectors preoperatively and 1 month postoperatively. The centroid shifted closer to the origin at 1 month post-surgery (0.21 D @ 111° ± 0.95 D) compared to the preoperative position (0.79 D @ 172° ± 2.12 D), accompanied by a reduction in the size of the 95% confidence ellipses. Vector analysis revealed a mean absolute difference of 1.98 ± 0.85 D.

### Optical quality

**Figure 3:**
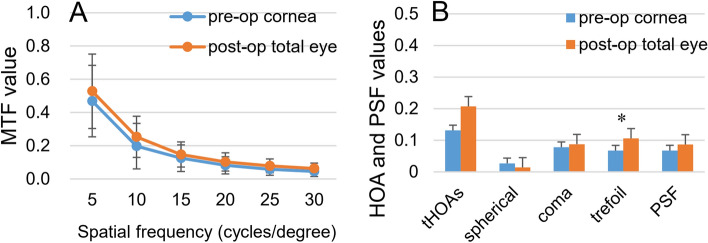
Optical quality of the preoperative cornea and the total eye postoperatively. (**A**) MTF values at spatial frequencies of 5 cpd, 10 cpd, 15 cpd, 20 cpd, 25 cpd, and 30 cpd (n = 11). There were no statistically significant differences between groups (*t* = − 0.661, − 0.921, − 0.610, − 1.007, − 1.382 and − 1.445, *P* = 0.523, 0.379, 0.556, 0.338, 0.197 and 0.179). (**B**) tHOAs, spherical aberration, coma, trefoil aberration and PSF values (n = 23). Postoperatively, the trefoil aberration of the total eye showed a significant reduction compared to that of the preoperative cornea (*Z* = 2.115, *P* = 0.034), while differences of tHOAs, spherical aberration, coma, and PSF values were not statistically significant (*Z* = − 1.776, *t* = 2.023, − 0.562 and − 0.827, *P* = 0.076, 0.054, 0.579 and 0.427). *MTF* modulation transfer function, *PSF* point spread function, *tHOAs* total higher-order aberrations, *cpd* cycles per degree, *P < 0.05.

Due to the interference of the opacified lens in the preoperative eye, we compared the optical parameters of the preoperative cornea with those of the total eye postoperatively. Postoperatively, the MTF values of the total eye (0.528 ± 0.215, 0.254 ± 0.137, 0.147 ± 0.080, 0.103 ± 0.052, 0.079 ± 0.036, 0.063 ± 0.031) showed mild improvement at spatial frequencies of 5 cycles per degree (cpd), 10 cpd, 15 cpd, 20 cpd, 25 cpd, and 30 cpd compared to the corresponding preoperative corneal MTF values (0.469 ± 0.224, 0.198 ± 0.124, 0.125 ± 0.076, 0.082 ± 0.055, 0.059 ± 0.042, 0.045 ± 0.033), but there were no statistically significant differences (*t* = − 0.661, − 0.921, − 0.610, − 1.007, − 1.382 and − 1.445, *P* = 0.523, 0.379, 0.556, 0.338, 0.197 and 0.179) (Fig. 3A). Postoperatively, the trefoil aberration of the total eye showed a significant reduction compared to that of the preoperative cornea (*Z* = 2.115, *P* = 0.034), while differences of tHOAs, spherical aberration, coma, and PSF values were not statistically significant (*Z* = − 1.776, *t* = 2.023, − 0.562 and − 0.827, *P* = 0.076, 0.054, 0.579 and 0.427) (Fig. 3B).

### Postoperative residual astigmatism prediction error

**Figure 4:**
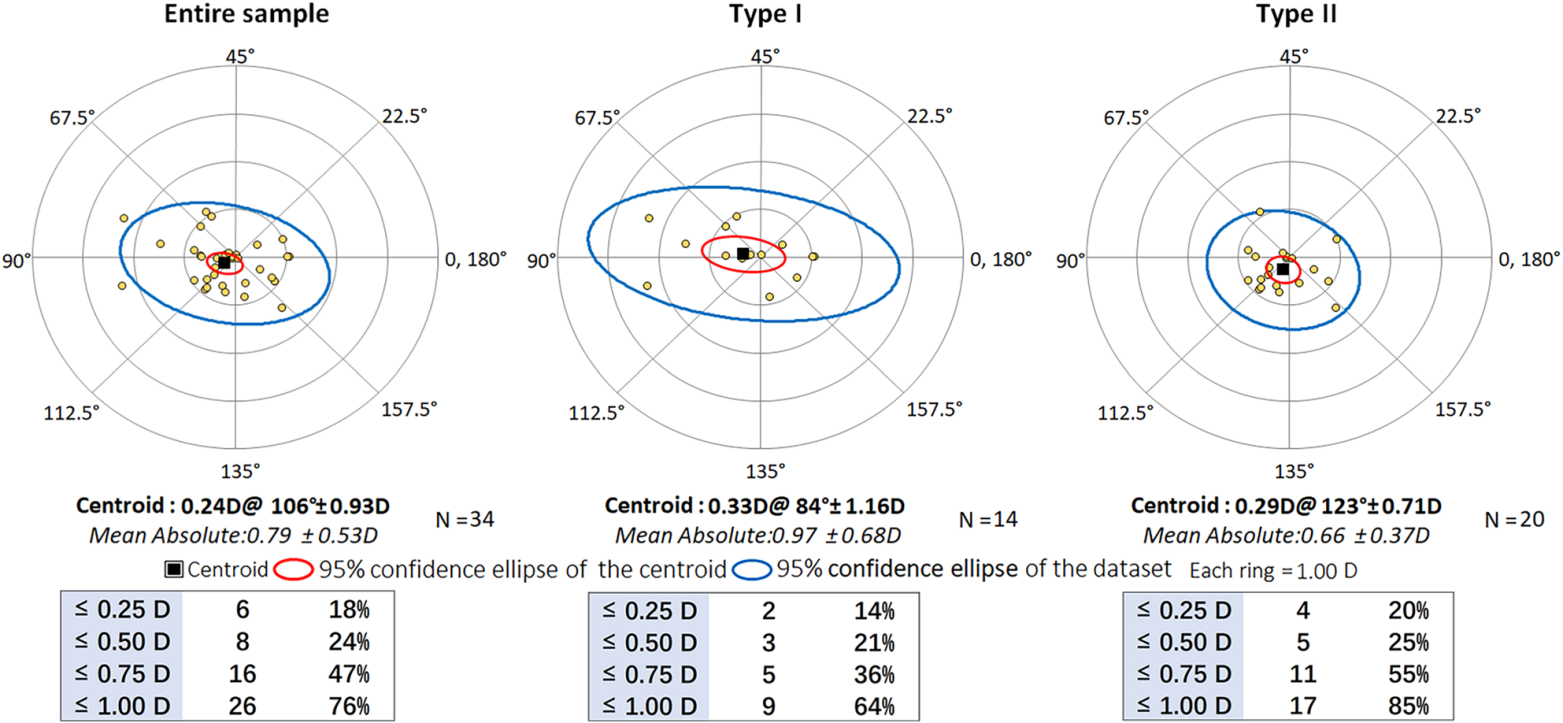
Double-angle plots and distribution of postoperative residual astigmatism prediction error. The mean absolute residual astigmatism prediction error of type II group (0.66 ± 0.37 D) was slightly smaller than those of type I group (0.97 ± 0.68 D), but the differences were not statistically significant (*t* = 1.694, *P* = 0.100). *D* diopters.

Vector analyses showed that the overall mean absolute postoperative residual astigmatism prediction error was 0.79 ± 0.53 D, with 24% below 0.50 D and 76% below 1.00 D. The centroid was 0.24 D @ 106° ± 0.93 D (Fig. 4). Subgroup analysis showed that the mean absolute residual astigmatism prediction error of type II group (0.66 ± 0.37 D) and its centroid (0.24 D @ 119° ± 0.72 D) were slightly smaller than those of type I group (0.97 ± 0.68 D and 0.33 D @ 84° ± 1.16 D), but the differences were not statistically significant (*t* = 1.694, *P* = 0.100).

**Figure 5:**
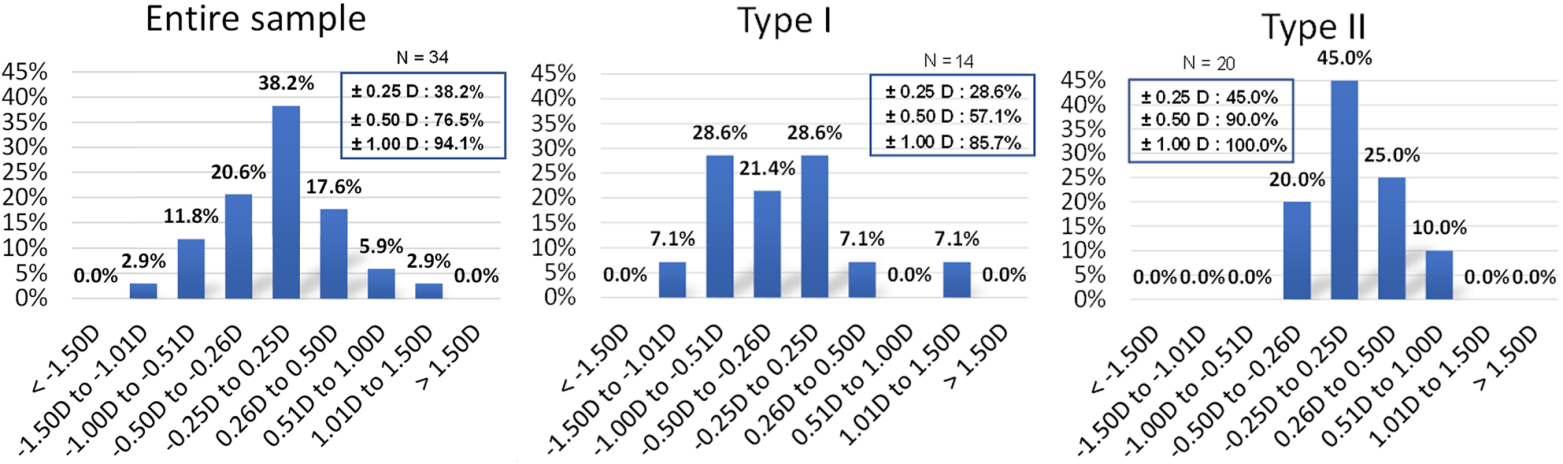
Distribution of SEQ prediction errors. The mean SEQ prediction error in type II group (0.07 ± 0.36 D) was significantly smaller than that in type I group (− 0.29 ± 0.52 D) (*t* = − 2.272, *P* = 0.030). *SEQ* spherical equivalent, *D* diopters.

Figure 5 illustrates the distribution of the SEQ prediction errors in the total, type I, and type II group. The mean SEQ prediction error in type II group (0.07 ± 0.36 D) was significantly smaller than that in type I group (− 0.29 ± 0.52 D) (*t* = -2.272, *P* = 0.030). Moreover, the distribution of errors in type II group was noticeably more concentrated, with 90.0% of cases having errors within ± 0.50 D, whereas that was only 57.1% in type I group.

## Discussion

This study demonstrates that cataract phacoemulsification combined with Toric IOLs implantation can effectively correct corneal irregular astigmatism to a significant extent and improve visual acuity. While there was no statistically significant difference in the predictive error of residual astigmatism between the two types of irregular astigmatism, we observed that cases classified as Type II demonstrated better accuracy in predicting SEQ compared to cases classified as Type I.

After implantation of the Toric IOL, the preoperative astigmatism decreased from an average of 2.05 ± 0.90 D (range: 1.08–5.24 D) to 0.78 ± 0.57 D (range: 0–2.43 D) postoperatively. There have been few reports on the use of Toric IOLs to correct irregular corneal astigmatism. In a study by Gao et al., among 23 eyes of irregular astigmatism with a regular central component, the preoperative corneal astigmatism was 1.99 ± 1.26 D (range: 1.15–6.97 D), and the residual postoperative astigmatism was 0.65 ± 0.57 D (range 0 to 2.75 D)^9^. Li et al. also achieved satisfactory results in their 30-eye-study from an average preoperative asymmetric bowtie corneal astigmatism of 2.18 ± 0.70 D to 0.72 ± 0.42 D postoperatively^10^. Kwitko et al. reported 88 eyes of asymmetric astigmatism or non-progressive corneal ectasia from an average of 2.32 ± 1.78 D preoperatively to 0.87 ± 1.09 D after Toric IOLs implantation^11^. All these studies are closely comparable to our findings (Supplementary Table S1). There are also a few relevant case reports regarding Toric IOLs implantation in irregular astigmatism cases caused by keratoconus (ranging from 2.00 to 4.62D), showing a certain effect of residual astigmatism ranging from 0.50 to 1.75D^7,12–14^.

Bogan et al. initiated a classification of corneal topography patterns using computer-assisted videokeratography, which were subsequently further subdivided into ten pattern types by Rabinowitz et al.^15^. In theory, not all patterns of irregular corneal astigmatism can be partially corrected by cylinders (such as the cylindrical component in Toric IOLs). Li et al.'s study exclusively included cases with the "asymmetric bow-tie" pattern^10^. Although Gao et al. included both the "asymmetric bow-tie" and "angled bow-tie" patterns, they did not compare them as we did^9^. Our study revealed that the correction outcomes of Type II cases, including postoperative residual astigmatism prediction error and SEQ prediction error, were superior to those of Type I cases. This could be attributed to the fact that most devices calculate the refractive power of the steep meridian by averaging the refractive powers of its hemimeridians (Supplementary Fig. S3). Consequently, in Type I cases, a regular Toric lens might overcorrect the side with a steeper slope and undercorrect the side with a flatter slope. Therefore, theoretically, an attempt could be made to directly utilize the refractive power of the flatter hemimeridian as the steep K for the Toric calculation. However, in cases classified as Type II, while the bow-tie pattern forms at a certain angle, the refractive powers on both hemimeridians are balanced, thus avoiding such issues. Through the observation of cases with varying steep meridian deviations (not 180°) or steep-flat meridian deviations (not 90°), Hwang et al. proposed that the larger the deviations, the worse the correction outcomes^16^. These characteristics provide valuable references for the clinical application of Toric IOLs in correcting irregular corneal astigmatism.

We found that, except for trefoil aberrations, Toric IOLs did not improve the optical quality defects caused by corneal irregularities. Li et al. discovered that tHOAs and coma were positively correlated with astigmatism prediction errors^10^. In the study by Gao et al., patients' optical quality, including modulation transfer function, strehl ratio, and objective scattering index, showed significant improvement. However, their assessment of the optical quality was about the entire optical system, primarily reflecting the effects of removing the opaque crystalline lens, rather than, as in our study, evaluating the correction of the cornea astigmatism by Toric IOLs implantation^9^.

While the cases in this study all presented with irregular corneal astigmatism, the measurements obtained from the IOL Master, iTrace, and Pentacam were consistent (i.e., astigmatism magnitude differed by no more than 1D, and steep meridian differences were within 10°). The consistency among multiple corneal measurement devices is crucial for achieving favorable outcomes in Toric IOLs utilization^17,18^, and this principle applies equally to cases with irregular corneal astigmatism. These different instruments operate on distinct principles and have slightly varying data capture ranges, which can further minimize the measurement errors due to corneal regularity. Additionally, it is beneficial to visually reassess the steep meridian multiple times using a manual keratometer^19^.

One limitation is that we only used standard K measurements obtained from the IOL Master 700 for Toric calculations, neglecting the measured posterior corneal astigmatism and the comparisons of different measurement devices (such as iTrace, Pentacam, anterior-segment optical coherence tomography, etc.) and different detection zones (e.g., corneal astigmatism derived from a specific zone). Another limitation is the lack of a control group with regular corneal astigmatism. Additionally, the relatively small sample size calls for cautious interpretation of the results.

In conclusion, our findings provide real-world evidence supporting the efficacy of Toric IOLs implantation in cataract surgery for eyes with specific types of irregular corneal astigmatism. Specifically, compared to cases classified as the "asymmetric bow-tie" pattern, postoperative refractive outcomes were more predictable in cases with "angled bow-tie" pattern. This offers these patients an alternative option besides corneal refractive surgery or corneal contact lens wear.

## Methods

### Patient enrollment

This retrospective cohort study was conducted in a tertiary hospital. The study was approved by the Ethics Committee of Tianjin Medical University Eye Hospital as exempt from informed consent and in accordance with the principles of the Helsinki Declaration (NO. 2020KY(L)-62). All patients provided written informed consent after receiving a detailed explanation about irregular astigmatism, Toric IOL implantation, and associated risks.

Patients with specific types of irregular corneal astigmatism who underwent uncomplicated phacoemulsification combined with Toric Acrysof IOL (model SN6ATx, *Alcon Laboratories, Texas, USA*) implantation were included in the study. Other inclusion criteria include: (1) Preoperative corneal astigmatism value measured by IOL Master 700 (*Carl Zeiss, Oberkochen, Germany*) ≥ 1.00 D. (2) Irregular corneal astigmatism diagnosed through the corneal topography map by Pentacam HR (*Oculus, Wetzlar, Germany*). (3) The corneal topography in the central 3-mm zone is displayed as a "bow-tie" pattern, and it should be represented by at least one of the following two patterns according to the classification proposed by Bogan et al.^20^ and Rabinowitz et al.^15^: (I) type I, the "asymmetric bow-tie" pattern, requires that the two main meridians be approximately orthogonal, with unequal slopes (with a K value difference more than 3.00 D) of the hemimeridians along a single meridian (Supplementary Fig. S1), (II) type II, the “angled bow-tie” or "symmetric bow-tie with skewed radial axes" pattern, requires hemimeridians of approximately equal slope but not aligned with each other (with an angle between 135° and 150°, Supplementary Fig. S2).

Exclude cases where the magnitude of corneal astigmatism obtained from IOL Master 700, Pentacam HR, and iTrace (*Tracey Technologies, Houston, TX, USA*) differs by more than 1.00 D, or where the steep meridian differs by more than 10°. Exclude irregular astigmatism types in corneal topography showing the two meridional lines (or their tangents) were not approximately vertically distributed, round, oval, or central irregular patterns. Patients with zonular weakness, corneal disease, history of intraocular surgery, and other ocular diseases that may affect visual function were also excluded from the study, as well as eyes with incomplete postoperative data at one-month follow-up.

### Preoperative examinations and IOL calculations

All patients underwent a comprehensive preoperative examination. The uncorrected distance visual acuity (UDVA) and best corrected visual acuity (BCVA) were measured by Phoropter. The biometric measurements including corneal refractive power were measured by IOL Master 700. Keratometric astigmatism derived from standard K measurements obtained by IOL Master 700 and topography of the corneal front obtained by Pentacam HR were used in this study. The modulation transfer Function (MTF), point spread function (PSF), and aberrations, including total higher-order aberrations (tHOAs), spherical aberration, coma, and trefoil aberration, were measured using iTrace at a pupil scanning diameter of 3 mm. The built-in Barrett Universal II formula in the IOL Master 700 was used to calculate the IOL power targeting emmetropia. The Barrett Toric Calculator (https://ascrs.org/tools/barrett-toric-calculator) was used to calculate the cylinder power and determine the axis orientation. The Toric IOL model with the minimum expected residual astigmatism was selected. All the parameters involved, including keratometry, axial length, anterior chamber depth, white-to-white, and lens thickness, were derived from IOL Master 700.

### Surgical technique

After adequate anesthesia, a preoperative cow-horn toric marker was used to make a horizontal mark along the corneal limbus in the 0°–180° meridian while the patient was seated and maintained a vertical head position. After adequate mydriasis, a 2.2 mm clear corneal incision was made at 135°. Conventional phacoemulsification procedure was performed. Intraoperative toric marker was used to mark the intended axis for implantation at the corneal limbus. The Toric IOL was implanted in the capsular bag and aligned with the intended axis. All operations were performed by the same experienced ophthalmologist (F. T.).

### Postoperative follow-up

The patients were followed up for 1 day, 1 week, and 1 month after surgery. The UDVA, BCVA, manifest refraction, MTF and HOAs at 1 month were recorded and used for statistical analyses. The residual astigmatism was derived from the postoperative refraction converted to the corneal plane at 1 month after surgery.

### Statistical analysis

All data were statistically analyzed using SPSS software (version 26; *IBM SPSS Statistics, Chicago, IL*, USA, https://www.ibm.com/docs/en/spss-statistics/26.0.0). Skewness, Kurtosis, and Normality were checked before data analysis. Comparisons of astigmatism, MTF, PSF, spherical aberration, and coma between preoperative and postoperative periods were conducted using paired t-tests, of which data were normally distributed. Due to the skewed distribution of the data, comparisons of tHOAs and trefoil were performed using the Wilcoxon Rank-Sum Test. Postoperative residual astigmatism and spherical equivalent (SEQ) prediction errors were calculated and plotted using Astigmatism Double Angle Plot Tool (version 1.3.2, https://ascrs.org/tools/barrett-toric-calculator), while their differences between the type I and type II irregular corneal astigmatism were compared using independent samples *t*-test (normally distributed). A *P*-value of < 0.05 was considered statistically significant.

### Supplementary Information

Supplementary Figure 1.

Supplementary Figure 2.

Supplementary Figure 3.

Supplementary Table 1.

## Data Availability

The datasets generated during and/or analysed during the current study are available from the corresponding author on reasonable request.
