# Supplementary material for: Effectiveness of toric intraocular lens implantation for correcting irregular corneal astigmatism in cataract eyes
Source: Sci Rep. 2024 Apr 17;14:8868. doi: 10.1038/s41598-024-59303-0 (PMC11024119; doi:10.1038/s41598-024-59303-0)
Supplement: Supplementary file 1 — Supplementary Figure 1. [file 41598_2024_59303_MOESM1_ESM.pdf]

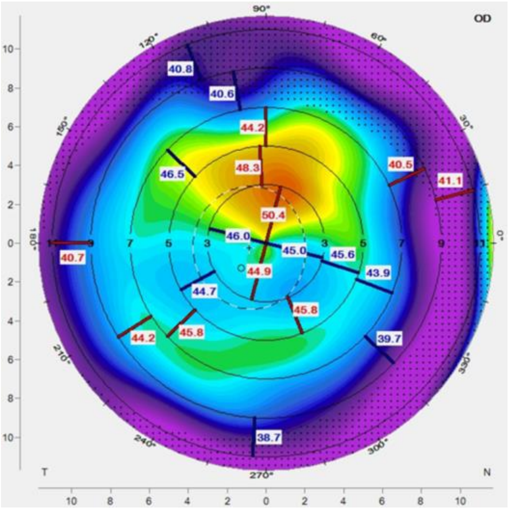

Supplementary Figure 1. Example of a corneal topography showing the "asymmetric bow-tie" pattern (type I). The two main meridians are approximately orthogonal, with unequal slopes (the two hemimeridians have a K value difference of more than 3.00 D) along a single meridian (50.4 D and 44.9 D in this case).
