# Supplementary material for: Effectiveness of toric intraocular lens implantation for correcting irregular corneal astigmatism in cataract eyes
Source: Sci Rep. 2024 Apr 17;14:8868. doi: 10.1038/s41598-024-59303-0 (PMC11024119; doi:10.1038/s41598-024-59303-0)
Supplement: Supplementary file 2 — Supplementary Figure 2. [file 41598_2024_59303_MOESM2_ESM.pdf]

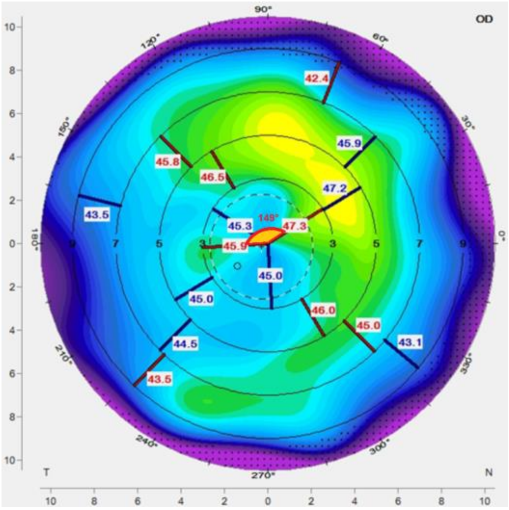

Supplementary Figure 2. Example of a corneal topography showing the "angled bow-tie" pattern (type II). The two hemimeridians have approximately equal slopes but are not aligned with each other (with an angle between 135 to 150 degrees, 149 degrees in this case).
