# Supplementary material for: Effectiveness of toric intraocular lens implantation for correcting irregular corneal astigmatism in cataract eyes
Source: Sci Rep. 2024 Apr 17;14:8868. doi: 10.1038/s41598-024-59303-0 (PMC11024119; doi:10.1038/s41598-024-59303-0)
Supplement: Supplementary file 3 — Supplementary Figure 3. [file 41598_2024_59303_MOESM3_ESM.pdf]

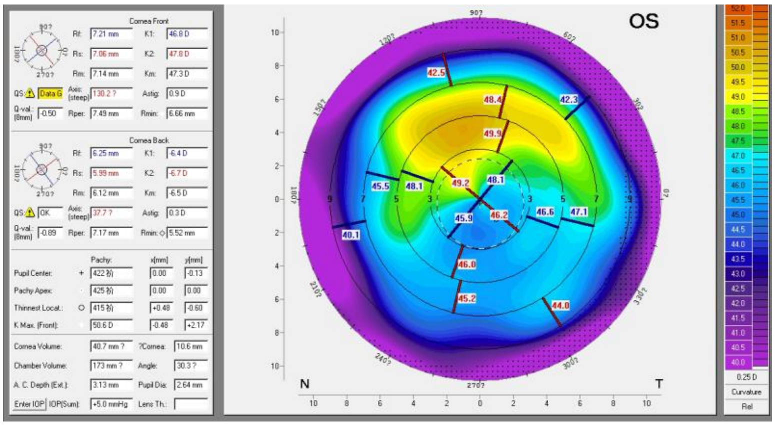

Supplementary Figure 3. Corneal topography and keratometry results of a case measured using Pentacam HR. The refractive power of the steep meridian (K2) in this case is calculated as 47.8 D, obtained by averaging the refractive powers of its two hemimeridians (49.2 D and 46.2 D).
