# Supplementary material for: Effectiveness of toric intraocular lens implantation for correcting irregular corneal astigmatism in cataract eyes
Source: Sci Rep. 2024 Apr 17;14:8868. doi: 10.1038/s41598-024-59303-0 (PMC11024119; doi:10.1038/s41598-024-59303-0)
Supplement: Supplementary file 4 — Supplementary Table 1. [file 41598_2024_59303_MOESM4_ESM.pdf]

Supplementary Table 1. Literatures on correcting irregular corneal astigmatism with Toric IOL implantation.

Studies / Years	Eyes / Patients	Type of astigmatism	Study design	Toric IOL	Preoperative corneal astigmatism (D)	Postoperative resisual astigmatism (D)	Postoperative SEQ (D)
Li H, et al/2023[12]	30/30	asymmetric bowtie	prospective cohort study	CT Asphina 709MP	2.18±0.70 (1.09–3.88)	0.72±0.42	/
Hwang HS, et al/2021[17]	112/78	irregular corneal steep and flat meridian	retrospective cohort study	Tecnis ZCT	2.21±1.36	0.57±0.31	/
Gao Y, et al/2020[9]	23/20	irregular astigmatism with a regular central component	prospective clinical observational study	AcrySof SN6AT2-9	1.99±1.26 (1.15–6.97)	0.65±0.57 (0–2.75)	/
Kwitko S, et al/2020[13]	88/69	asymmetric astigmatism and non-progressive corneal ectasia	retrospective cohort study	Acrysof SN60TT; Rayner T-Flex; Tecnis ZCT; Zeiss AT-Torbi	2.32±1.78 (0.25–8.00)	0.87±1.09 (0–5.50)	0.85±3.36 (0–6.50)
Allard K, et al/2018[7]	1/1	keratoconus	case report	AcrySof SN6AT9	4.62	1.75	2.40
Navas A, et al/2009[15]	2/2	keratoconus	case report	AcrySof SN60TT5/4	3.10/2.0	0.50/0.50	0.50/0
Kolozsvári BL, et al/2017[16]	1/1	keratoconus	case report	AcrySof SN6AT6	2.5	0.75	1.75
Visser N, et al/2011[14]	3/2	keratoconus	case report	Acrysof SN60T9/9/5	4.1/3.7/2.1	1.50/1.50/0.75	0.75/3.25/0.75

D = diopters, SEQ = spherical equivalent
